# Hip screw lateral migration with no cut-out or non-union implication: a case report

**DOI:** 10.1186/1757-1626-2-6419

**Published:** 2009-03-10

**Authors:** Nikolaos Lasanianos, Georgios Mouzopoulos, Ioannis Georgilas

**Affiliations:** 1 1st Trauma and Orthopaedic surgery department, "Evangelismos" General Hospital of Athens, Lontou 12, Palea Penteli, 15236, Athens-Greece; 2 1st Trauma and Orthopaedic surgery department, "Evangelismos" General Hospital of Athens, Sofokli Venizelou 23, Peristeri 12131, Athens-Greece; 3 1st Trauma and Orthopaedic surgery department, "Evangelismos" General Hospital of Athens, Ypsilantou 45, 10676, Athens-Greece

## Abstract

Hip screw migration of peritrochanteric fracture fixation devices is a described complication in English literature. Medial migration occupies the majority of these cases whereas lateral migration is rare. We report the case of an 85-year-old woman whose intramedullary osteosynthesis of a trochanteric fracture was complicated by hip screw lateral migration. Mobilization was not influenced and no cut-out or non-union was detected. The migrated hip screw was easily removed and the discomfort vanished. The need for adequate surgical technique and radiographic examination after re-injuries even if the patient remains ambulatory is emphasised.

## Introduction

Fractures of the trochanteric region of the femur are very common in the elderly. Many fixation devices have been developed for these fractures; the most widely used being the numerous versions of intramedullary nails which tend to replace the sliding nail plate systems. Intra-operative blood loss and operating time is much minimised, immediate load-bearing is ensured, and postoperative morbidity remains low [1]. Despite the good and reliable results, some typical failures and complications may occur [2,3].

One of the most common complications of intramedullary systems is lag screw migration combined with hardware cut-out or non-union [4,5]. Herein we present the rare case of a laterally migrated hip screw of an intramedullary device after the fixation of a peritrochanteric fracture. Up to our knowledge this is the first case of a lateral migration in a single hip screw fixation device, without an accompanying non-union or cut-out.

## Case presentation

**Figure 1:**
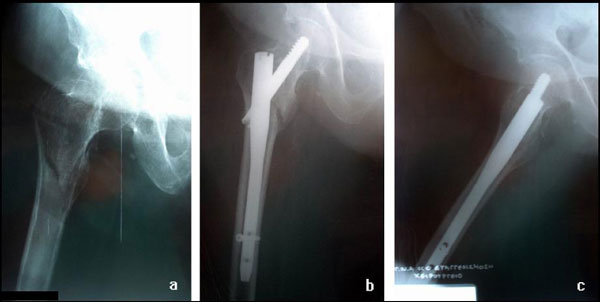
**a A type 31-A.** 2.2 left hip intertrochanteric fracture; **b** Postoperative anteroposterior view of the fracture shown on figure 1a; **c** Postoperative lateral view of the fracture shown on figure **1a**.

**Figure 2:**
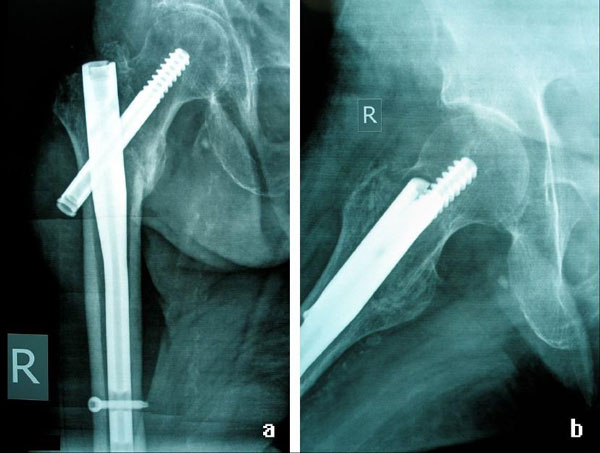
**a 2**.5 months post surgery anteroposterior view showing a slight lag screw backsliding of about 10 mm; **b** 2.5 months post surgery lateral view showing a slight lag screw backsliding of about 10 mm.

**Figure 3:**
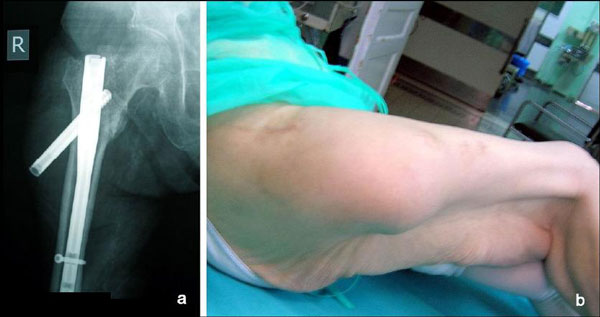
**a 3 months post surgery anteroposterior radiographic view showing full lateral migration of the hip screw after a new fall**; **b** Clinical view of the swollen right hip.

**Figure 4:**
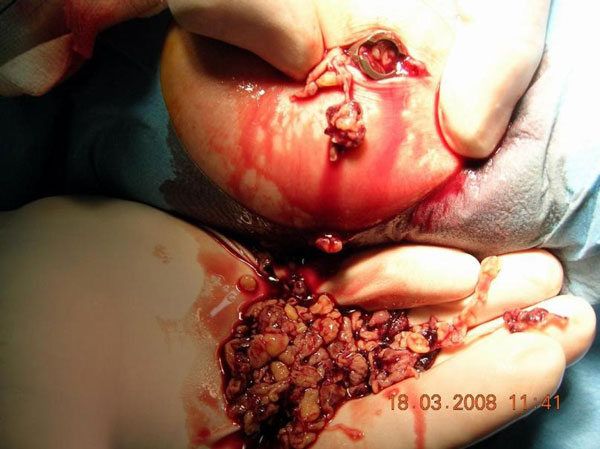
**Intra-operative photo of the hip screw removal showing the amount of post-traumatic haematoma and molten fats**.

An 85-year-old woman presented with a hip fracture after a fall. The radiographic control revealed a type 31-A.2.2, secondary to the AO classification, intertrochanteric fracture (Figure 1a) of the right femur. The fracture was fixed internally with a 130° angle, 180 mm length ATN Trochanteric nail (DePuy, Johnson & Johnson Company). A 100 mm length and 10.5 mm diameter lag screw was used and distal dynamic locking was performed. Reaming was performed to the femoral head before the placement of the lag screw and to the medullary canal before the insertion of the nail. Although the ATN system provides an optional anti-rotation screw (placed above the lag screw into the femoral head) this was not used. Postoperative x-rays showed very good fracture reduction. The Garden alignment index was 160° in the anteroposterior (AP) view (Figure 1b) and 180° in the lateral view (Figure 1c) which consist the absolute desirable values. The lag screw position was ideal as seen in the post-op AP and lateral view. The Tip Apex Distance (TAD) was considered to be no more than 10 mm. Partial weight bearing was advised for 2 weeks postoperatively and then full weight bearing was allowed. The patient's mobilization program was developing normally without pain restriction or other walking difficulties. Scheduled radiographic control performed 2.5 months post surgery (Figure 2a &2b) revealed a slight lag screw backsliding of about 10 mm. At that point full callus formation was achieved and the patient was advised not to restrain from her daily activities. Two weeks after the last examination the patient sustained a fall. A new radiographic control revealed a total lateral migration of the lag screw (Figure 3a). No radiographic signs of cut-out, re-fracture or non-union were noticed. Swelling of the right hip was noticed the following days (Figure 3b) and the patient started complaining of night pain during bed time. Despite these findings she remained ambulatory and did not face any problems with her mobilization. The migrated lag screw was removed under local anaesthesia on the basis of one day surgery about one month after the new injury. During the screw removal drainage of about 300 ml of post-traumatic haematoma and molten fats from the subcutaneous hip was performed (Figure 4). The nail was left in place and the wound was closed.

## Discussion

Peritrochanteric fractures represent a significant risk in every age group. Moreover in the elderly they may represent a risk to life. Intramedullary nailing is widely used for fixation of such fractures ensuring less operating time, minimized wounds, immediate weight bearing, faster mobilization and thus less morbidity [1]. Nevertheless complications never cease existing.

The most common and well-documented mechanical complication of these devices are: a) cut-out of the hip screw through the femoral head with varus collapse of the fracture [6]; b) fracture of the femoral shaft distal to the tip of the implant, a phenomenon reduced by the newer nail designs [7]; c) medial migration of the hip screw into the pelvis which is more rarely reported. The latter has been termed the "Z-effect" and has been described primarily for two-screw devices such as the proximal femoral nail (PFN, Synthes, Switzerland) [8]. Nevertheless, it has occurred in implants with a single femoral head fixation element as well, including the trochanteric fixation nail (TFN), Gamma, Zimmer, and Depuy nails [3,9]-[12].

Lateral migration is more rare than medial. Up to our knowledge there are not any published cases of lateral migration, in a single use of a hip screw, without a new fracture or a cut-out accompanying the migration. Studies concerning the use of perhaps the most studied nail (Gamma nail - Howmedica) conclude that there have been no instances of implant failure without non-union or re-fracture [13].

Hip screw migration is considered to be an evolutionary complication and the ability of the implant to resist migration under dynamic loading is of critical importance. Walking subjects the implant-bone interface to combined axial and torsional loading and may play a role in lag screw migration. In our case slight, painless lateral migration (of approximately 10 mm) was noticed 2.5 months post surgery and full lateral migration was noticed two weeks later after the occurrence of a new fall. The new injury caused great nail protrusion and haematoma of the hip accompanied with tenderness which gave away after the lag screw removal. We could not reach any profound explanation for the migration mechanism. The fact that the way of the lag screw into the femoral head was reamed and that the diameter of the non-threaded part of the lag screw was the same with that of the thread could provide some explanation for the behaviour of the screw and more particularly for the convenience of total migration once it was slightly detached from its original position. Moreover the osteoporotic bone of the patient did not offer an adequate grip to stabilize the sliding screw and prevent its loosening. Osteoporosis on the other hand did not seem to create any delay to bone union.

One could suppose that this is the case of a semi-completed Z-effect since the lag screw migrated laterally as if the optional anti-rotational screw (which we did not use) was there to play the role of the medially migrated half. It should be reminded here that Werner-Tutschku et al [8] were the first to describe the so called Z-effect phenomenon as the medial migration of the antirotation screw with the simultaneous lateral migration of the lag screw. The nature and aetiology of the Z-Effect phenomenon has not yet been identified. As hypothesis, a helical rotation of the antirotation screw but also an axial migration due to jerky micromotions under weight-bearing are discussed. A caput-collumn-diaphysis (CCD) angle below 125°, osteoporosis and several attempts at reaming represent risk factors for the Z-effect phenomenon. A further explanation is the impactation of the hip pin into the proximal hole of the nail while the neck screw normally slides back during the weight-bearing period [12]. In our case it could be supposed that the non placement of the anti-rotational screw resulted in slight rotational instability of the lag screw. This may have caused the slight lateral migration that was first noticed and was completed after the mechanical impaction of the new fall.

The age of the patient, the quality of the bone, the pattern of the fracture, the stability of the reduction, the angle of the implant, and the position of the lag screw within the femoral head have all been related to this mechanism of failure, but there has been no clear consensus as to the interrelationships or relative importance of each factor. Previous clinical studies suggest that lag screws placed with a tip-apex distance (TAD) of less than *25* mm should rarely fail by cut-out [14,15]. In our case this was achieved since the TAD was measured to be less than 10 mm. This might have been the determinant factor that resulted in early callus formation and thus relevant protection of the fractured area from lag screw micro motions and protrusion, avoiding re-fracture, non-union or excessive mechanical stress of the device.

## Conclusion

Intramedullary nailing tends to become the treatment of choice for proximal femoral fractures and has a low implant failure rate. Although closed reduction and minimally invasive exposure minimise delayed union and non-union they are not enough for the avoidance of such complications. Caution is needed in the placement of the lag screw in manner that respects the limitations of Tip Apex Distance. A well centred lag screw contributes to better transmission of axial forces and inducts the earlier callus formation. Thus complications such the one described in this paper do not result in re-fractures, non-union or mal-union.

## Abbreviations

AP: Anteroposterior; PFN: Proximal Femoral Nail; TAD: Tip Apex Distance; TFN: Trochanteric Fixation Nail; CCD: Caput Collumn Diaphysis.

## Consent

Written informed consent was obtained from the patient for publication of this case report and accompanying images. A copy of the written consent is available for review by the Editor-in-Chief of this journal.

## Competing interests

The authors declare that they have no competing interests.

## Authors' contributions

NL collected the data and drafted the manuscript. GM involved in revising the manuscript for important intellectual content. IG was the consultant surgeon responsible for the patient. All authors read and approved the final manuscript.
